# Tacrine(10)-Hupyridone Prevents Post-operative Cognitive Dysfunction via the Activation of BDNF Pathway and the Inhibition of AChE in Aged Mice

**DOI:** 10.3389/fncel.2018.00396

**Published:** 2018-11-07

**Authors:** Huixin Chen, Xiang Wu, Xinmei Gu, Yiying Zhou, Luying Ye, Ke Zhang, Hanbo Pan, Jialing Wang, Hua Wei, Binbin Zhu, C. Benjamin Naman, Shinghung Mak, Paul R. Carlier, Wei Cui, Yifan Han

**Affiliations:** ^1^Zhejiang Provincial Key Laboratory of Pathophysiology, Ningbo Key Laboratory of Behavioral Neuroscience, School of Medicine, Ningbo University, Ningbo, China; ^2^Department of Anesthesia, Ningbo University Medical School Affiliated Hospital, Ningbo, China; ^3^Li Dak Sum Yip Yio Chin Kenneth Li Marine Biopharmaceutical Research Center, Ningbo University, Ningbo, China; ^4^State Key Laboratory of Chinese Medicine and Molecular Pharmacology (Incubation), The Hong Kong Polytechnic University Shenzhen Research Institute, Shenzhen, China; ^5^Department of Applied Biology and Chemistry Technology, Institute of Modern Chinese Medicine, The Hong Kong Polytechnic University, Hong Kong, China; ^6^Department of Chemistry, Virginia Tech, Blacksburg, VA, United States

**Keywords:** tacrine(10)-hupyridone, post-operative cognitive dysfunction, brain-derived neurotrophic factor, acetylcholinesterase, choline acetyltransferase

## Abstract

Post-operative cognitive dysfunction (POCD) could cause short-term or long-term cognitive disruption lasting weeks or months after anesthesia and surgery in elderly. However, no effective treatment of POCD is currently available. Previous studies indicated that the enhancement of brain-derived neurotrophic factor (BDNF) expression, and the elevation the cholinergic system, might be effective to prevent POCD. In this study, we have discovered that tacrine(10)-hupyridone (A10E), a novel acetylcholinesterase (AChE) inhibitor derived from tacrine and huperzine A, could prevent surgery-induced short-term and long-term impairments of recognition and spatial cognition, as evidenced by the novel object recognition test and Morris water maze (MWM) tests, in aged mice. Moreover, A10E significantly increased the expression of BDNF and activated the downstream Akt and extracellular regulated kinase (ERK) signaling in the surgery-treated mice. Furthermore, A10E substantially enhanced choline acetyltransferase (ChAT)-positive area and decreased AChE activity, in the hippocampus regions of surgery-treated mice, indicating that A10E could prevent surgery-induced dysfunction of cholinergic system, possibly via increasing the synthesis of acetylcholine and the inhibition of AChE. In conclusion, our results suggested that A10E might prevent POCD via the activation of BDNF pathway and the inhibition of AChE, concurrently, in aged mice. These findings also provided a support that A10E might be developed as a potential drug lead for POCD.

## Introduction

Post-operative cognitive dysfunction (POCD) is widely observed in the elderly in post-operative periods, resulting in the increase of medical care, patient burden and mortality (Smith et al., 2018). POCD causes alterations of learning, memory and behavior that can last for weeks or months after anesthesia and surgery, leading to short-term or long-term cognitive disruption (Rasmussen and Steinmetz, 2015). It is therefore urgent to develop pharmacological treatments for this disorder. Unfortunately, the pathophysiology of POCD remains unclear, and no effective drug is available. Therefore, it is urgent to develop pharmacological treatments for this disease.

Previous studies have shown that neurotrophic factors, including brain-derived neurotrophic factor (BDNF) in particular, are down-regulated in the brains of aged rodents subjected to anesthesia and surgery (Zhang et al., 2014; Zhao et al., 2017). The level of acetylcholine in the hippocampus is also decreased in aged rats after anesthesia and surgery, correlating with the impairments of cognitive functions (Su et al., 2012; Wu et al., 2017). It is thus possible that chemicals owing the ability to enhance the expression of BDNF or elevate the concentration of acetylcholine might be potential candidate for the treatment of POCD.

Acetylcholinesterase (AChE) is an enzyme that decomposes acetylcholine into acetate and choline, and is present in the synaptic clefts in the brain. The use of AChE inhibitors elevates the concentration of acetylcholine in treated tissues, and can have the ability to enhance cognitive performance (Brousseau et al., 2007). A recent study has shown that donepezil could reverse cognitive impairments in aged rodents subjected to anesthesia and surgery (Zhang et al., 2018). Moreover, there are many clinical trials evaluating the effects of FDA-approved AChE inhibitors, such as rivastigmine and donepezil, on the prevention and treatment of POCD^1^. Although the results of these clinical trials are not revealed yet, this information provides a support that AChE inhibitors might be helpful to treat POCD.

We have previously designed and synthesized a series of dimers derived from tacrine and huperzine A, two AChE inhibitors used for treating Alzheimer’s disease (AD) in clinical (Li et al., 2007). Among these dimers, tacrine(10)-hupyridone (A10E), is most potent to inhibit AChE with an IC_50_ around 20 nM (Li et al., 2007). Moreover, since both tacrine and huperzine A can increase the expression of BDNF in the brain, it is suggested that A10E might inhibit AChE and elevate BDNF expression concurrently (Zhao et al., 2011; Mao et al., 2016).

We have previously established a pre-clinical POCD model induced by abdominal surgery under anesthesia in mice at the age of 12 months (Wu et al., 2017). In the present study, we have evaluated the effects of A10E on surgery-induced cognitive impairments using this established model. We have also investigated the underlying molecular mechanisms of A10E against POCD.

## Materials and Methods

### Chemicals and Reagents

Fentanyl (fentanyl citrate injection, 0.05 mg/mL) was obtained from Humanwell Pharmaceutical Co., Ltd. (Yichang, Hubei, China). Droperidol (droperidol injection, 2.5 mg/mL) was purchased from Sun Rise Pharmaceutical Co. Ltd (Shanghai, China). A10E was synthesized as previously described by us (Li et al., 2007).

### Animal Experiments

Male ICR mice at age 12 months and weighing 30–40 g were supplied by Zhejiang Academy of Medical Sciences. Mice were exposed to a 12 h light/dark cycle under controlled humidity (50 ± 10%) and temperature (22 ± 2°C). Four to five animals were kept in each cage. Animals were given free access to normal animal food (Shanghai Slac Laboratory Animal Co., Ltd., Shanghai, China) and water. All procedures were performed according to the guidelines recommended by the National Institutes of Health (NIH) Guide for the Care and Use of Laboratory Animals (NIH Publications No. 80-23, revised 1996) and approved by the Animal Care and Use Committee of Ningbo University. The number of the approval for the animal experiments was SYXK-2013-0191.

Forty mice were randomly allocated to five groups, each containing eight mice, as follows: control, surgery and surgery plus 0.2, 0.4 and 0.6 mg/kg A10E (dissolved in saline). Mice in the surgery group were treated with normal saline as the vehicle. Drugs or vehicle were administered by intraperitoneal (i.p.) injection once daily for 32 consecutive days.

Mice were given i.p. injections with a mixture of fentanyl (0.02 mg/kg) and droperidol (5.0 mg/kg). The blink reflexes of the mice were used to determine anesthetic effects. All surgeries were performed by a standardized procedure. Briefly, the fur of the mice in the surgical site was shaved. A laparotomy was conducted using a 1.5 cm midline incision. Approximately 5 cm of the small intestine was removed from the peritoneal cavity, then covered with clean and moist gauze. After 3 min, the small intestine was replaced in the peritoneal cavity, and two layers of the abdominal wall were closed by sutures. All the procedures lasted approximately 10 min.

**Figure 1:**
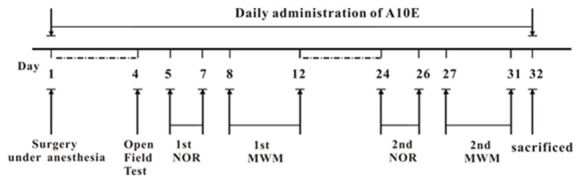
Design of animal experiments. Day 1, mice were administrated with a mixture of 0.02 mg/kg fentanyl and 5.0 mg/kg droperidol to cause anesthesia. Then, a laparotomy was conducted to induce post-operative cognitive dysfunction (POCD). Each day, tacrine(10)-hupyridone (A10E; 0.2–0.6 mg/kg) was injected intraperitoneally (i.p). for 32 days. After surgery, mice were allowed to recover for 4 days. At day 4 post-operation, the motor functions of mice were evaluated by the open field test. The effect of A10E on recognition and spatial cognition was tested by novel objective recognition (NOR) or morris water maze (MWM) testing at each of two phases on days 5–12 and days 24–31 post-operation, respectively. On the final day, animals were sacrificed.

The whole procedure of behavioral tests is displayed in Figure 1. Briefly, at day 4 post-surgery, the motor functions of the mice were evaluated by open field tests. On days 5–7 and 8–12 post surgery, the 1st novel objective recognition (NOR) and Morris water maze (MWM) tests were used to measure recognition and spatial cognition in the early stage, respectively. On days 24–26 and 27–31 post surgery, recognition and spatial cognition in the late stage were further evaluated by the 2nd NOR test and MWM tests, respectively.

### NOR Test

The NOR test was conducted in an open-field arena (30 × 30 × 30 cm) constructed with polyvinyl chloride, plywood and acrylic, as described previously (Bevins and Besheer, 2006). The task included acclimation, training and retention over three consecutive days. On the 1st day, the animals were acclimated to the experimental area for 5 min without exposure to any behavioral stimuli. On the 2nd day, the animals explored two identical objects (black plastic cubes, 5 × 5 × 5 cm) for 5 min. On the 3rd day, one of the objects was replaced with a new shape and color (a gray plastic square pyramid, 5 × 5 × 7 cm), and the animals were again acclimated to the area for 5 min. The field was decontaminated with 70% ethanol solution and dry cloth between the tests. The animals explored the test area by sniffing or touching the objects with their nose and/or forepaws at a distance of less than 2 cm. Sitting on or turning around the objects was not considered exploratory behavior. The exploratory behavior of each animal was evaluated by an observer blinded to the test conditions, after recording using a video camera. Total exploration time refers to the amount of time devoted to the location of the two objects. Cognitive function was measured using a recognition index, which is the exploration time involving either of the two objects (training session) or the novel object (retention session) compared with the total exploration time.

### MWM Test

Spatial memory was tested using the MWM as described previously (Morris, 1981). The water maze comprised a circular pool measuring 110 cm in diameter, filled with water at 23 ± 2°C to immerse a platform. The platform was always positioned in the middle of the northwest quadrant except on the last day. Swimming was recorded by a video camera linked to a computer-based imaging system. Learning was evaluated for four consecutive days. Each mouse was trained to locate the platform during four trials per day, and the time required to reach the hidden platform was measured. On the 5th day, a probe trial was conducted by removing the platform and training the mice to swim for 90 s to locate it. Swimming time in the four quadrants of the pool was calculated. Preference for a previous quadrant occupied by the platform indicated spatial memory.

### Brain Tissue Harvest

Animals were deeply anesthetized and perfused transcardially with ice-cold saline. Brains were surgically removed very quickly. Proteins were extracted for Western blot analysis (four mice per group) and stored at −80°C before use. Other brain tissues (four mice per group) were used for immunofluoresence staining and AChE activity measurements.

### Western Blot

Western blot was conducted as described previously (Cui et al., 2014). Briefly, brain tissue was extracted at 4°C for 1 min using a lysis buffer, and centrifuged at 16,000 *g* for 10 min. The protein levels in the supernatant were estimated by the Bradford assay, followed by SDS-PAGE of tissue samples (40 μg), and transfer to polyvinylidene fluoride membrane. The membranes were blocked with 5% non-fat milk in TBST for 2 h, and incubated overnight at 4°C with primary antibodies against pAkt, Akt, pERK, extracellular regulated kinase (ERK) and β-actin (Cell Signaling Technology, Beverly, MA, USA). After washing the samples three times with TBST, the membranes were incubated with a secondary antibody. Blots were developed using enhanced chemiluminescence as instructed by the manufacturer (Amersham Bioscience, Aylesbury, UK). All data were representative of three independent experiments. Data were expressed as ratios of optical density (OD) compared with controls for statistical analyses.

### Immunofluoresence Staining

Briefly, after the behavioral tests, brains were dissected and incubated with 4% paraformaldehyde for 24 h. After rinsed with 0.1 M phosphate buffer (PBS) and dried, the brain tissues were dehydrated in 30% glucose solution for 48 h until sinking to the bottom. Then the specimens were cut into 20 μm sections on a freezing microtome. Sections from regions containing the hippocampal tissue were processed for BDNF and choline acetyltransferase (ChAT) staining, which were transferred into multi-well plate containing 1–2 mL PBS. The sections were incubated with 1:100 diluted primary BDNF (Abcam, UK) and ChAT (Santa Cruz, CA, USA) antibodies at 4°C overnight. The specimens were rinsed and incubated with fluorescence secondary antibodies (Solarbio, China) for 60 min in dark. The sections were labeled with 4′,6-diamidino-2-phenylindole (DAPI) and observed using a fluorescence microscope. The images were digitized and quantified using ImageJ. ImageJ was then used to enhance the image contrast, apply a threshold to distinguish positively stained features from the background, and apply a size exclusion threshold to identify and distinguish BDNF and ChAT. The protocol was modified according to a previous publication (Koga et al., 2013).

### Measurement of AChE Activity *ex vivo*

The assay of AChE activity measurement was modified from previous study (Mak et al., 2014). Briefly, brains of mice collected immediately after decapitation were used as the source of AChE. Brain was weighted and added 10 times volume of lysis buffer (10 mM HEPES, pH 7.5, 1 mM EDTA, 1 mM EGTA, 150 mM NaCl and 0.5% Triton X-100). The homogenization was done by vortexing on ice for 15 min. The homogenates were clarified by centrifugation for 15 min at 3,000 rpm at 4°C. The assay medium contained 0.1 M Na_2_HPO_4_ (pH 7.5), 10 mM DTNB and 1 mM ATCI. The brain lysate was incubated with 0.1 mM ethopropazine hydrochloride for 5 min to inhibit butyrocholinesterase activity. Test compounds were added to the assay solution and pre-incubated at 37°C with the enzyme for 15 min followed by the addition of substrate. The activity was determined by measuring the absorbance at 412 nm after incubation at 37°C for 30 min.

### Data Analysis

Non-parametric one-way ANOVA was analyzed by using Kruskal-Wallis test. If there is statistical significance among groups, Dunn’s multiple comparison test was used for *post hoc* multiple comparisons. Significant differences were accepted at *p* < 0.05.

## Results

### Neither Surgery Nor A10E Significantly Changed Motor Functions of Mice

**Figure 2:**
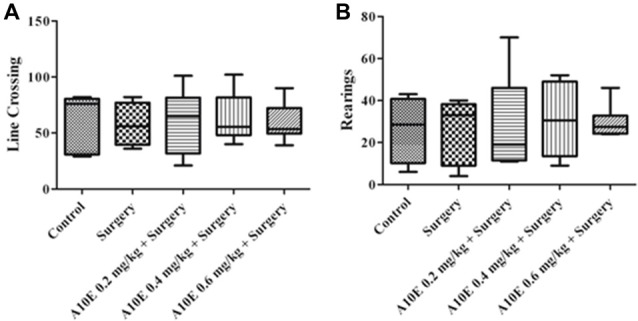
Treatments did not significantly change motor functions of mice after surgery. The open field test was performed on day 4 post-operation. The number of line crossing and rearing events were calculated, and the results are shown in **(A,B)**, respectively (*n* = 8).

To examine whether A10E or surgery could affect motor function of aged mice, the open field test was used. As demonstrated in Figure 2, treatments did not significantly alter the number of line crossing or rearings in the open field test in aged mice, suggesting that neither surgery nor A10E significantly affected motor function of the mice in this study (Kruskal-Wallis test, for line crossing, *p* = 0.962; for rearings, *p* = 0.952, Figure 2).

### A10E Significantly Prevented Surgery-Induced Impairments of Recognition and Spatial Cognition in the Early Stage Post Surgery

**Figure 3:**
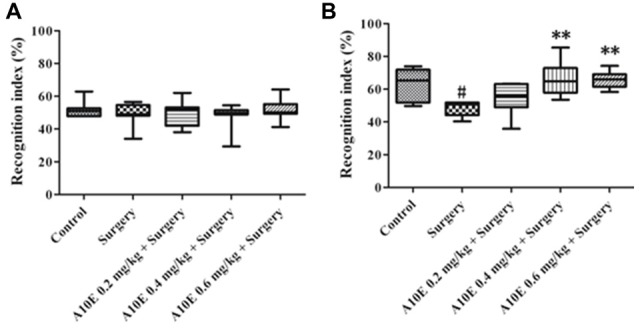
A10E significantly prevented surgery-induced impairment of recognition in the early stage. The 1st NOR test was performed on days 5–7 post-operation. **(A)** In the training session of the 1st NOR test, treatments did not significantly change the recognition index in aged mice. **(B)** In the retention session of the 1st NOR test, A10E treatment at 0.4–0.6 mg/kg significantly prevented the reduction of recognition index in surgery-treated mice (*n* = 8). ^#^*p* < 0.05 vs. the control group, **p < 0.01 vs. the surgery group (Dunn’s multiple comparison test).

In the early stage post surgery, the NOR and MWM tests were used to measure recognition and spatial cognition, respectively. In the training session of NOR test in the early stage, the recognition index among various groups was similar (Kruskal-Wallis test, *p* = 0.860, Figure 3A). However, in the retention session of the NOR test, the recognition index was significantly different (Kruskal-Wallis test, *p* = 0.001, Figure 3B). The recognition index in the control group was significantly higher than that in the surgery group (Dunn’s multiple comparison test, *p* < 0.05, Figure 3B). Moreover, A10E at 0.4–0.6 mg/kg significantly increased the recognition index (Dunn’s multiple comparison test, *p* < 0.01, Figure 3B).

**Figure 4:**
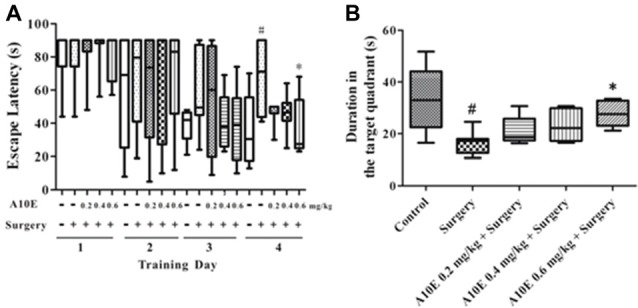
A10E significantly prevented surgery-induced impairment of spatial learning and memory in the early stage. The 1st MWM test was performed on days 8–12 post operation. **(A)** In the training trials of the 1st MWM test, treatment with A10E at 0.6 mg/kg significantly reduced the escape latency at the last day of test in surgery-treated mice. **(B)** In the probe trial session of the 1st MWM test, A10E treatment at 0.6 mg/kg significantly increased the time duration spent by animals in the target quadrant in surgery-treated mice (*n* = 8). ^#^*p* < 0.05 vs. the control group, *p < 0.05 vs. the surgery group (Dunn’s multiple comparison test).

In the training trials of the MWM test in the early stage post surgery, the escape latency was significantly different among groups in day 4, but not day 1–3 (Kruskal-Wallis test, for day 1, *p* = 0.997; for day 2, *p* = 0.941; for day 3, *p* = 0.353; for day 4, *p* = 0.024). Furthermore, on the 4th day of the training trials, the escape latency of mice in the surgery group was significantly higher than those in the control group (Dunn’s multiple comparison test, *p* < 0.05, Figure 4A). Mice treated with 0.6 mg/kg A10E spent significantly shorter time finding the platform when compared with mice in the surgery group (Dunn’s multiple comparison test, *p* < 0.05, Figure 4A). The spatial memory was also evaluated in the probe trial of the MWM in the early stage. The swimming duration in the target quadrant was significantly changed among various groups (Kruskal-Wallis test, *p* = 0.003, Figure 4B). Specifically, the duration of the surgery group swimming in the target quadrant was significantly shorter than that of the control group (Dunn’s multiple comparison test, *p* < 0.05, Figure 4B). Treatment with A10E at 0.6 mg/kg significantly increased the swimming duration the mice spent in the target quadrant (Dunn’s multiple comparison test, *p* < 0.05, Figure 4B).

### A10E Prevented Surgery-Induced Impairments of Recognition and Spatial Cognition in the Late Stage Post Surgery

**Figure 5:**
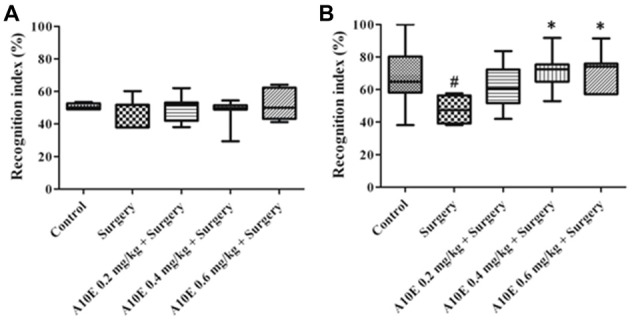
A10E significantly prevented surgery-induced impairment of recognition in the late stage. The 2nd NOR test was performed on days 24–26 post-operation. **(A)** In the training session of the 2nd NOR test, treatments did not significantly change the recognition index in aged mice. **(B)** In the retention session of the 2nd NOR test, treatment with A10E at 0.4–0.6 mg/kg significantly prevented the reduction of recognition index in surgery-treated mice (*n* = 8). ^#^*p* < 0.05 vs. the control group, *p < 0.05 vs. the surgery group (Dunn’s multiple comparison test).

In the late stage post-surgery, similar tests as above were used. The recognition index was similar among various groups in the training session of the NOR test in the late stage (Kruskal-Wallis test, *p* = 0.933, Figure 5A). In the retention session, the recognition index was different among various groups (Kruskal-Wallis test, *p* = 0.010, Figure 5B). The recognition index in the surgery group was lower than that in the control group (Dunn’s multiple comparison test, *p* < 0.05, Figure 5B). Furthermore, treatment with A10E at 0.4–0.6 mg/kg significantly reversed surgery-induced reduction of the recognition index (Dunn’s multiple comparison test, *p* < 0.05, Figure 5B).

**Figure 6:**
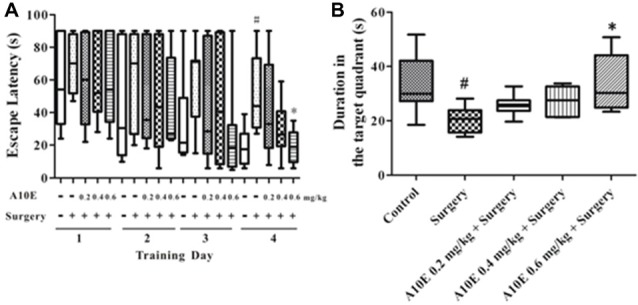
A10E significantly prevented surgery-induced impairment of spatial learning and memory in the late stage. The 2nd MWM test was performed on days 27–31 post operation. **(A)** In the training trials of the 2nd MWM test, treatment with A10E at 0.6 mg/kg significantly reduced the escape latency at the last day of testing in surgery-treated mice. **(B)** In the probe trial session of the 2nd MWM test, A10E treatment at 0.6 mg/kg significantly increased the time duration spent of animals in the target quadrant in surgery-treated mice (*n* = 8). ^#^*p* < 0.05 vs. the control group, *p < 0.05 vs. the surgery group (Dunn’s multiple comparison test).

**Figure 7:**
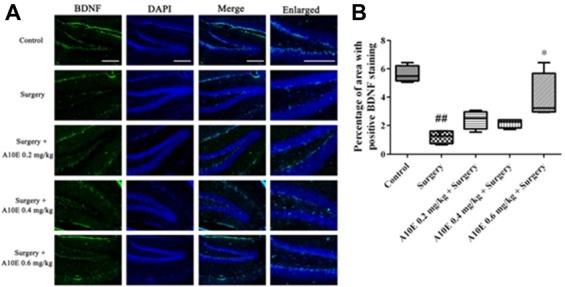
A10E significantly increased the brain-derived neurotrophic factor (BDNF)-positive area in the hippocampal regions of surgery-treated mice. **(A)** Representative images of BDNF staining in the hippocampal region of mice in various groups, as indicated (scale bar = 150 μm). **(B)** Quantitative results demonstrated that treatment with A10E at 0.6 mg/kg significantly increased BDNF-positive area in surgery-treated mice (*n* = 4). ^##^*p* < 0.01 vs. control group, *p < 0.05 vs. the surgery group (Dunn’s multiple comparison test).

For the results in the training trials of the MWM test in the late stage, the escape latency was significantly different among groups in day 4, but not day 1–3 (Kruskal-Wallis test, for day 1, *p* = 0.875; for day 2, *p* = 0.842; for day 3, *p* = 0.320; for day 4, *p* = 0.008). On the last day of the training trials, mice in the surgery group spent significantly longer times to find the platform than those in the control group (Dunn’s multiple comparison test, *p* < 0.05, Figure 6A). Treatment with 0.6 mg/kg A10E significantly decreased the escape latency of the mice finding the platform (Dunn’s multiple comparison test, *p* < 0.05, Figure 6A). At the probe trial of the MWM in the late stage post surgery, the swimming duration in the target quadrant was different among various groups (Kruskal-Wallis test, *p* = 0.010, Figure 7B). Surgery significantly decreased the swimming duration in the target quadrant (Dunn’s multiple comparison test, *p* < 0.05, Figure 6B). Treatment with A10E at 0.6 mg/kg significantly increased the swimming duration in surgery-treated mice (Dunn’s multiple comparison test, *p* < 0.05, Figure 6B).

### A10E Significantly Increased the Expression of BDNF in the Hippocampi of Surgery-Treated Mice

The expression of BDNF in the hippocampi of mice was analyzed by immunofluoresence staining (Figure 7A). The BDNF-positive area was different among various groups (Kruskal-Wallis test, *p* = 0.003, Figure 7B). The BDNF-positive area in the control group was significantly greater than that in the surgery group (Dunn’s multiple comparison test, *p* < 0.05, Figure 7B). Treatment with A10E at 0.6 mg/kg significantly prevented the surgery-induced decrease of BDNF expression detected after staining (Dunn’s multiple comparison test, *p* < 0.05, Figure 7B), suggesting that A10E increased the expression of BDNF in the hippocampi of surgery-treated mice.

### A10E Significantly Enhanced the Expression of pAkt and pERK in Surgery-Treated Mice

**Figure 8:**
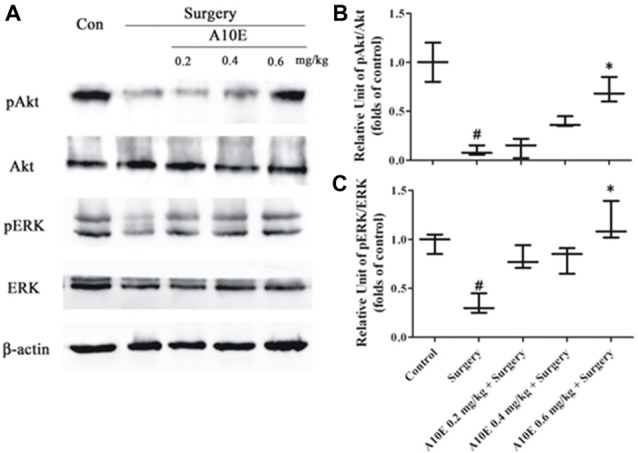
A10E significantly increased the expression of pERK and pAkt in the hippocampal regions of surgery-treated mice. **(A)** The expression of pAkt, Akt, pERK, extracellular regulated kinase (ERK) and β-actin in the hippocampal regions of mice was detected by Western blotting. Quantitative results demonstrated that treatment with A10E at 0.6 mg/kg significantly increased the expressions of **(B)** pAkt and **(C)** pERK in surgery-treated mice (*n* = 4). ^#^*p* < 0.05 vs. control group and *p < 0.05 vs. the surgery group (Dunn’s multiple comparison test).

BDNF activates both Akt and ERK pathways (Leal et al., 2014). Therefore, we further evaluated the expression of pAkt and pERK in test animals by using Western blotting analysis, since these are the key molecules that regulate the Akt and ERK pathways. It was demonstrated that the expression of levels pAkt and pERK were significantly higher in the control group than those in the surgery group (Dunn’s multiple comparison test, *p* < 0.05, Figure 8). Moreover, treatment with A10E at 0.6 mg/kg reversed the surgery-induced decrease of pAkt and pERK expression (Dunn’s multiple comparison test, *p* < 0.05, Figure 8). These results suggested that A10E activated the Akt and ERK pathways in surgery-treated mice.

### A10E Significantly Enlarged ChAT-Positive Area in the Hippocampi of Surgery-Treated Mice

**Figure 9:**
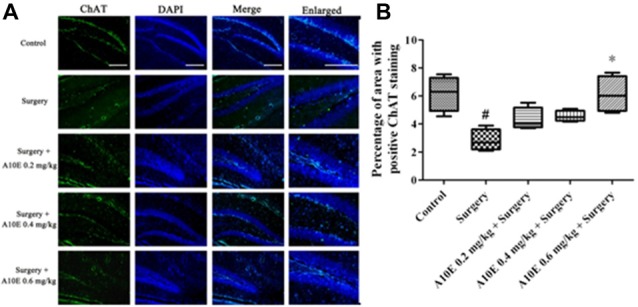
A10E significantly increased the choline acetyltransferase (ChAT)-positive area in the hippocampal regions of surgery-treated mice. **(A)** Representative images of ChAT staining in the hippocampal region of mice in various groups, as indicated (scale bar = 150 μm). **(B)** Quantitative results demonstrated that treatment with A10E at 0.6 mg/kg significantly increased ChAT-positive area in surgery-treated mice (*n* = 4). ^#^*p* < 0.05 vs. control group, *p < 0.05 vs. the surgery group (Dunn’s multiple comparison test).

The expression of ChAT in the hippocampi of mice was analyzed by immunofluorescence staining (Figure 9A). The ChAT-positive area for the surgery group was significantly smaller than that in the control group (Dunn’s multiple comparison test, *p* < 0.05, Figure 9B). Moreover, treatment with A10E at 0.6 mg/kg significantly reversed the surgery-induced decrease of ChAT-positive area (Dunn’s multiple comparison test, *p* < 0.05, Figure 9B), indicating that A10E could enlarge the ChAT-positive area in surgery-treated mice.

### A10E Decreases Surgery-Induced Increase of AChE Activity *ex vivo*

**Figure 10:**
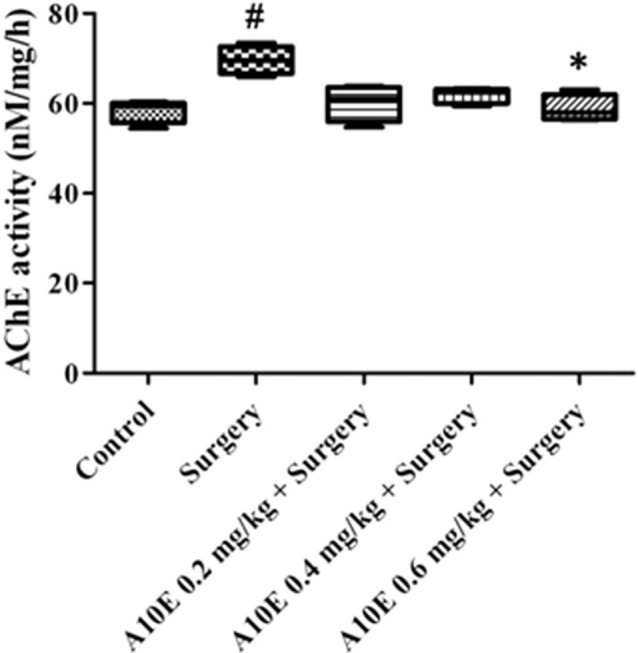
A10E significantly prevented surgery-induced inhibition of acetylcholinesterase (AChE) activity *ex vivo*. A10E was injected 30 min before the sacrifice. AChE activity were measured *ex vivo* (*n* = 4). ^#^*p* < 0.05 vs. the control group, *p < 0.05 vs. the surgery group (Dunn’s multiple comparison test).

AChE activities in the hippocampus were also analyzed *ex vivo*. The activity of AChE was different among various groups (Kruskal-Wallis test, *p* = 0.019, Figure 10). A10E at 0.6 mg/kg significantly prevent surgery-induced increase of hippocampal AChE activity* ex vivo* (Dunn’s multiple comparison test, *p* < 0.05, Figure 10).

## Discussion

In this study, we have discovered that A10E, an AChE inhibitor derived from tacrine and huperzine A, prevents some surgery-induced impairments of recognition and spatial cognition in aged mice. We further found that A10E was able to increase the expression of BDNF, activate the Akt and ERK pathways, elevate the ChAT-positive area, and decrease the activity of AChE in the hippocampi of surgery-treated mice, which might partially explain the observed effects of A10E on cognitive enhancement.

Although many factors can affect the severity of POCD, the type of surgery, anesthetics used and the age of patients are widely considered to be the key factors involved. Previous studies have shown that peripheral surgery under anesthesia could induce cognitive impairments in aged patients (Müller et al., 2016; Skvarc et al., 2018). Therefore, in mice, we chose exploratory laparotomy under anesthesia to mimic peripheral surgery under anesthesia in patients. We used 12-month-old mice, which are generally considered as aged mice (Wang et al., 2016). We also used a fentanyl-droperidol combination that is traditionally used to induce anesthesia (Zanette et al., 2010). Most importantly, we have previously reported that such an exploratory laparotomy under anesthesia induced cognitive impairments in aged mice, suggesting that this model might be useful to test the effects of chemicals on the prevention of POCD (Wu et al., 2017).

To further evaluate the effects of A10E on short-term and long-term cognitive impairments, we used the NOR and MWM tests, two reliable and classical behavioral tests for exploring recognitive and cognitive performance, respectively, in rodents. NOR is a non-force driving and spontaneous memory test for evaluating recognition, which is derived from curiosity. MWM is supposed to measure spatial memory and cognitive mapping based on the factor that mice are natural swimmers, but dislike water. In the early (1–15 days) and the late (16–32 days) stages post-surgery, which mimic the short-term and long-term impairments in aged patients after surgery, respectively, the recognitive and cognitive performance of mice in the surgery-treated group was significantly worse than that of mice in the control group. These results agree well with the results of a previous study that showed exploratory laparotomy under anesthesia could induce both short-term and long-term recognitive and cognitive impairments in aged rodents (Wang et al., 2016).

AChE inhibitors are used clinically to treat neurological diseases associated with cognitive impairments (Galimberti and Scarpini, 2016). In this study, A10E at 0.4–0.6 mg/kg effectively prevented surgery-induced recognitive impairments by using NOR tests, However, A10E only at 0.6 mg/kg could prevent surgery-induced learning and memory impairments in MWM test, suggesting that the surgery-induced cognitive impairments might be harder to be prevented than recognition impairments by A10E in mice. Interestingly, A10E at 0.6 mg/kg could prevent both early and late stage learning and memory impairments in mice, providing a support that A10E might be able to prevent short-term and long-term impairments of learning and memory after surgery in aged patients.

Previous studies have shown that AChE inhibitors could improve cognitive performance via elevating cholinergic signaling (Galimberti and Scarpini, 2016). In our study, A10E significantly prevent surgery-induced elevation of AChE activity *ex vivo*, indicating that A10E, like other AChE inhibitors, could improve cognitive and recognitive performance via acting on cholinergic signaling in rodents. Furthermore, it could be that besides AChE inhibition, A10E acts on other biological targets to exert its function in the brain. BDNF was an important neurotrophic factor, regulating not only learning and memory, but also stress and motivation (Li and Wolf, 2015). Previous studies have shown that surgery in aged rodents largely reduced the expression of BDNF in the hippocampus of brain, leading to the decrease of synaptic plasticity and neuronal loss (Fan et al., 2016; Wei et al., 2018). In this study, A10E was shown to prevent surgery-induced decrease of BDNF in the hippocampus of aged mice, indicating that A10E might act on BDNF to enhance cognitive performance. These results are consistence with reports that other tacrine and huperzine A derived compounds are able to elevate the expression of BDNF, and provided a support that A10E might also prevent surgery-induced damage of neurons and synapses (Zhao et al., 2011; Mao et al., 2016).

BDNF can bind to TrkB, its receptor in the neurons, and activate the signaling pathways of Akt and ERK (Leal et al., 2014). To further investigate whether A10E could affect these two signaling pathways, the Western blotting assay was used to analyze the expression of pAkt and pERK. The results showed that A10E increased the expression of pAkt and pERK, and provided support that A10E might act on BDNF and its downstream signaling pathways.

Long-term administration of AChE inhibitors can regulate dysfunction of the cholinergic system in animals. ChAT is an enzyme mainly located in cholinergic neurons to produce acetylcholine. Therefore, ChAT is regularly used as a marker of cholinergic neurons (Hut and Van der Zee, 2011). In this study, A10E significantly increased ChAT-positive area in the hippocampal regions of surgery-treated mice, which suggested that A10E could prevent surgery-induced dysfunction of the cholinergic system, possibly via inhibition of AChE and by elevating BDNF, concurrently. We speculated that for the short-term impact on surgery-induced impairments, A10E mainly inhibits AChE to elevate cognitive performance. For the long-term impact, A10E could activate BDNF signaling, reserve cholinergic neurons, and inhibit AChE, concurrently, leading to the prevention of surgery-induced damage and the enhancement of cholinergic signaling.

In addition, our study provides a connection between POCD and cholinergic signaling, Shenhar-Tsarfaty et al. (2016) have reported that AChE is increased with the aging, indicating the association between aging and cholinergic decline. Moreover, inhibiting cholinergic signaling in aged population could cause the increased risk of dementia (Tuszynski et al., 2015). Both studies supported our finding that enhancing cholinergic signaling by A10E may be useful to treat POCD.

However, the present study has several limitations to be addressed. First, the toxicity of A10E needs to be evaluated in follow-up experiments, since this was not here determined. Tacrine itself has been discontinued in clinical use due to its hepatotoxicity, so this must be considered whether A10E can also produce hepatotoxicity remains unknown. However, the dosing concentrations of A10E (0.4–1.2 μmol/kg) used in this study were much lower compared to those for tacrine (8–12 μmol/kg) when enhanced cognitive performance, suggesting that the risk of toxicity with A10E use might also be less than that for tacrine (Han et al., 2012). Second, the detailed mechanisms of action by which A10E modulates BDNF expression were not explored in this study. Micro RNA is a small non-coding RNA that functions in post-transcriptional regulation of gene expression. Many studies have shown that miRNA, such as miR-132 and miR-204, play important roles in the regulation of BDNF and cholinergic signaling, neural activity and cognitive performance (Qian et al., 2017). It is possible that A10E modulates BDNF and ChAT expressions via acting on miRNA. To prove this point, additional experiments are needed.

In conclusion, it is shown here that A10E, a new AChE inhibitor, prevented surgery-induced cognitive impairments in aged mice. It was also found that A10E treatment elevated the expression of BDNF, activated the Akt and ERK signaling pathways, prevented dysfunction of cholinergic system, and inhibited AChE activity, which all might contribute to improved cognitive performance. Based on these findings, it is suggested that A10E might be further developed as a potential drug lead for POCD.

## Author Contributions

WC, YH and XW contributed to the conception and design of the study. HC, XG, YZ, LY, KZ, HP, HW and JW performed the experiments and statistical analysis. HC, BZ, SM and PC wrote the first draft of the manuscript. All authors contributed to manuscript revision, read and approved the submitted version.

## Conflict of Interest Statement

The authors declare that the research was conducted in the absence of any commercial or financial relationships that could be construed as a potential conflict of interest.

## Footnotes

^1^http://clinicaltrials.gov
